# Accelerate the process of getting vaccinated: factors associated with consideration of and accessibility to COVID-19 vaccination in metropolises of China

**DOI:** 10.1186/s12889-022-13567-1

**Published:** 2022-06-14

**Authors:** Yinliang Tan, Zhilan Xie, Ying Qian, Jie Gu, Yundan Bai, Xiaoqing Gu, Zheng Ye, Jianmin Feng, Jiaoling Huang

**Affiliations:** 1 School of Public Health, Shanghai Jiao Tong University School of Medicine, 227 S Chongqing Rd, Huangpu District, Shanghai, 200025 China; 2 Business School, University of Shanghai for Science and Technology, Shanghai, China; 3 Department of General Practition, Zhongshan Hospital Fudan University, Shanghai, 200093 China; 4 Chengdu First People’s Hospital, Health Management Medical Center, Chengdu, Sichuan Province China; 5 Xidu Community Health Service Center of Fengxian District, Shanghai, China; 6 Changfeng Community Health Service Center of Putuo District, Shanghai, China; 7 Department of General Practition, First Hospital of Nanping, Nanping, Fujian Province China

**Keywords:** COVID-19 pandemic, Vaccination, Health policy, Health disparity

## Abstract

**Background:**

Rapid mutation of the severe acute respiratory syndrome coronavirus 2 (SARS-CoV-2) virus is sweeping the world and delaying the full reopening of society. Acceleration of the vaccination process may be the key element in winning the race against this virus. We examine factors associated with personal considerations of and accessibility to the corona virus disease 2019 (COVID-19) vaccination in metropolises of China.

**Methods:**

This multi-center, cross-sectional research was conducted using online questionnaires from April 1 to June 1, 2021, in community health service centers of Shanghai, Chengdu and Fuzhou. 9,047 vaccinated participants were included and data for 8,990 individuals were eligible for analysis. Chi-square test was conducted to find potential predictors, which were included in the logistic regressions. The odds ratios (ORs) and 95% confidence intervals (CIs) were calculated to assess the influence of region, socio-economic status (SES), and attitudes on vaccination process.

**Results:**

In consideration phase, participants in Fuzhou (OR:2.26, 95%CI: 1.90 to 2.68) and Chengdu (OR: 2.48, 95%CI: 2.17 to 2.83) were more likely than those in Shanghai to consider longer than one month. These odds increased for participants with master or above degree (reference: illiteracy and primary school), higher monthly household income (reference: < ¥5000), and greater vaccination hesitancy (reference: low hesitancy). Unemployed and household-based participants (OR: 3.37, 95%CI: 1.69 to 6.75, reference: farmer) and participants without brand preference (OR:1.13, 95%CI:1.02 to 1.26) may take longer time of consideration. In the accessibility phase, participants in Fuzhou (OR: 8.82, 95%CI: 7.28 to 10.68) and Chengdu (OR: 2.28, 95%CI: 1.98 to 2.63) were more likely to wait longer than one week. These odds decreased for participants with master or above degree (reference: illiteracy and primary school), monthly household income from ¥5000 to ¥10,000 (reference: < ¥5000), and teacher or student (reference: farmer). Participants without brand preference (OR: 0.86, 95%CI: 0.77 to 0.95) were likely to wait shorter after appointment, while participants with higher risk awareness of domestic epidemic (medium, OR: 1.24, 95%CI: 1.12 to 1.37, reference: low) may wait longer.

**Conclusions:**

The influential factors changed over two phases of vaccination process. Regional disparity affected both consideration and accessibility phases. Expect that, SES, and hesitancy were major factors of the consideration phase, but had limited impact on accessibility phase.

## Background

Several severe acute respiratory syndrome coronavirus 2 (SARS-CoV-2) variants have now been detected across the globe, having spread to more than 160 countries as of August, 2021 [1]. Concerns about the reduction in vaccine efficacy have, moreover, arisen globally. Fortunately, recent studies have demonstrated vaccination effectiveness of around 67% to 88% against symptomatic diseases caused by variants of concern [2], and significantly reduced infectivity in vaccinated cases [3, 4]. Research has also found that low vaccination coverage for corona virus disease 2019 (COVID-19), rather than loss of vaccine protection, contributes to SARS-CoV-2 mutation, as most of the variants have occurred in less vaccinated populations [5]. This is a wake-up call for countries: the need to combat virus mutation by promoting domestic vaccination is extremely urgent.

However, as of the beginning of 2022, only 5.5% of people in low-income countries had been fully vaccinated, compared with 72% of the population in high-income countries [6]. As part of efforts to promote vaccinations, extensive research worldwide has explored factors associated with vaccination uptake; at a micro-level, personal attitudes—especially vaccination acceptance and hesitancy—played an important role in decision making [7, 8] and have been widely explored for COVID-19 vaccination programs [9–11]. The evidence also showed that poorer vaccination rates occurred among lower socio-economic status (SES) groups [12, 13]. In addition, economic development, socio-cultural factors, and health system responses differed across regions, accounting for remarkable regional vaccine disparities [14–16].

The vaccination process can be divided into two phases: the consideration phase and the accessibility phase. However, most studies have not distinguished between the two phases and have not explored their respective influential factors, which may differ widely [17, 18]. Additionally, we also explored these factors by region in view of the uneven regional development in China. Three selected metropolises presented large cities with high population density and frequent population flows, which can cause rapid spread of virus. In sum, this study sought to identify the crucial factors for each phase separately, relating to COVID-19 vaccination, and on the basis of our findings, to propose appropriate interventions to accelerate vaccination rates around the world.

## Methods

### Study design

This multi-center, cross-sectional survey was conducted in three metropolises: Shanghai, Chengdu (capital city of Sichuan province), and Fuzhou (capital city of Fujian province). The cities were selected according to the China’s city-tier classification [19], a widely accepted categorization system first published by the Chinese news source Yicai Global. This classification is carried out based on five aspects (concentration of commercial resource, hub position, citizen vitality, lifestyle diversity and future plasticity). One city was randomly selected from each tier: Tier 1 (Shanghai), emerging Tier 1 (Chengdu), and Tier 2 (Fuzhou). The survey included adults who were vaccinated against COVID-19 in China between April 1, 2021 and June 1, 2021, and excluded children, adolescents (< 18 years old), elderly (> 65 years old), and pregnant or lactating women, owing to possible contraindications. The minimum sample size for each city was calculated for statistical significance (according to the formula below).

$$\mathrm{n}= \frac{{\mathrm{Z}}_{\mathrm{\alpha }/2}^{2}(1-\mathrm{p})}{{\upvarepsilon }^{2}\mathrm{p}}* (1+20\mathrm{\%}) = \frac{{1.96}^{2}*(1-0.20)}{{0.1}^{2}*0.2}*1.2 = 1844$$

where n denotes the sample size for each city, z the value for α = 0.025 in each tail, p estimated prevalence (since the distribution of consideration and accessibility phase in vaccinated population are unknown in previous studies, we used the lowest prevalence of outcome variables in sample of three cities), $\upvarepsilon$ the acceptable margin of error, 20% the estimate of the highest unqualified rate.

Ethical approval was granted by Fengxian District Central Hospital medical ethic committee (2021-ethic approve-02) and written informed consent was obtained from each participant before they began the survey. This study followed the Strengthening the Reporting of Observational Studies in Epidemiology (STROBE) reporting guidelines for cross-sectional studies and all procedures were carried out in accordance with the provisions of the World Medical Association Declaration of Helsinki (2013).

### Data collection

We adopted a multi-stage stratified systematic sampling method. Three representative districts (downtown, suburban, and rural) were selected from each city, and two community health service centers (CHSCs) were randomly selected from each district, resulting in a total of 18 CHSCs. We recruited investigators in each city, who then received online training for investigation. The survey was conducted in the waiting room of CHSCs where the residents should stay for 30 min after getting vaccinated, supported by the general practitioners (GPs) and nurses in the selected CHSCs. Any person whose vaccination ID ended with 8 on the survey day was invited to participate in the survey, and to provide informed consent. If a participant was unfamiliar with digital questionnaires or lacked digital equipment, the investigator provided them with a printed version; completed paper surveys were input daily by Epi-data. The online questionnaires would not be submitted before answering all questions and off-line version would be checked by investigators before accepted to avoid missing data. The survey initiated from April in three cities, and we collected the data for a full week in every round to ensure the representativeness of sample, considering that vaccinations were unevenly distributed throughout one week (some people came for vaccinations mainly on weekends, especially workers and students). Data collection ended when the number of participants in each city reached the minimum sample size. Altogether, 9,047 participants were recruited.

### Measurement

The entire vaccine process was divided into two phases. The first, labeled the consideration phase, referred to the period beginning when a participant heard about the available COVID-19 vaccine and ending when they made an appointment to be vaccinated; the second, or accessibility phase, covered the time from making the vaccination appointment to the point at which the participant received their first vaccine dose. Each phase was divided into two categories with a cut-off point at the median: ≤ 1 month (coding “0”) or > 1 month (coding “1”) for the consideration phase, and ≤ 1 week (coding “0”) or > 1 week (coding “1”) for the accessibility phase. The independent variables included a regional factor (i.e., Shanghai, Chengdu, or Fuzhou), SES (i.e., education level, occupation, and monthly household income), and personal attitudes towards COVID-19 and vaccines (i.e., vaccine brand preference, vaccination hesitancy, and risk awareness for the domestic epidemic). Other basic information comprised age, sex, marriage status, disability status, and whether had contracted with a GP. Some categories in our questionnaire like SES were developed based on the classification of several Chinese official researches [20–22]. Besides, previous researches about vaccination attitudes and behaviors also contributed to the formation of questionnaire contents [13, 23–25], in consideration of specific situation (like general practitioners) and public concerns in China.

### Statistical analysis

All analyses were performed using IBM SPSS version 24.0 and R version 4.1.0. The final sample size was 8,990 (eligible rate = 99.37%) after removing those who did not pass the data quality check (n = 57), such as the logic problem for mutually exclusive items and those who chose the same option for over 70% questions. Basic information was presented as a number with a percentage or a mean with a standard deviation (SD). Age was tested (t-test) and proved to be a significant variable. In addition, chi-square analysis was performed for other personal information and independent variables in both phases. Of the variables tested in the chi-square analysis, those that were significant in at least one phase were selected as potential predictors and finally included in the multivariate logistic regressions, as shown in Table 2. At last, the odds ratios (ORs) and 95% confidence intervals (CIs) were calculated to determine whether region, SES, and/or attitudes affected the vaccination process. Statistical significance was set at a two-sided *p* < 0.05 level.

## Results

### General description of participants

Of the 8,990 eligible participants included in the survey analysis, 3,788 (42.14%) were vaccinated in Shanghai, 2,258 (25.12%) were vaccinated in Fuzhou, and 2,944 (32.75%) were vaccinated in Chengdu (see Table 1). Participants’ mean age was 29.55 (SD = 11.63), most were male (55.98%), unmarried (59.21%), non-disabled (98.87%), white-collars or students (67.91%) and had graduated from university or junior college (63.16%). Most (75.45%) reported a monthly household income of ¥20,000 or less. The sample was representative of general population in terms of gender and income.

**Table 1:** Characteristics of study participants in Shanghai, Fuzhou, and Chengdu (*n* = 8990)

Characteristics	n(%) or Mean ± SD
Place	
Shanghai	3788(42.14)
Fuzhou	2258(25.12)
Chengdu	2944(32.74)
Age	29.55 ± 11.63
Sex	
Male	5033(55.98)
Female	3957(44.02)
Marriage	
Unmarried	5323(59.21)
Married	3278(36.46)
Divorce	224(2.49)
Widow/widower	26(0.29)
Else	139(1.55)
Education	
Illiteracy and primary school	107(1.19)
Junior high school	1038(11.55)
Senior high school	1232(13.70)
University and junior college	5678(63.16)
Master and above	935(10.40)
Occupation	
Farmer	170(1.89)
Civil servant	147(1.64)
Teacher	327(3.64)
Medical staff	104(1.16)
White-collar	2081(23.15)
Student	4024(44.76)
Worker	723(8.04)
Freelance work	536(5.96)
Housework and unemployment	64(0.71)
Else	814(9.05)
Monthly Income in family (¥)	
< 5000	2342(26.05)
> = 5000 and < 10,000	2758(30.68)
> = 10,000 and < 20,000	1683(18.72)
> = 20,000 and < 50,000	1074(11.95)
> = 50,000	1133(12.60)
Disability	
Yes	102(1.13)
No	8888(98.87)

### Factors associated with each vaccination phase

#### Distribution and univariate analysis of examined factors

Table 2 presents the distribution and the univariate analysis of the possible factors relevant in the vaccination process. Some of the factors associated with both consideration and accessibility phases were statistically significant; they were region, education, occupation, brand preference, vaccination hesitancy, and domestic risk awareness (*p* < 0.05). Disability status (p = 0.001) and GP (*p* < 0.001) were associated only with the consideration phase, while household income (p = 0.002) difference was observed in the accessibility phase.

**Table 2:** The distribution of factors associated with time for phase I, phase II, and the entire vaccination process

Variable n(%)	Consideration phase			Accessibility phase		
	< = 1 m	> 1 m	*P* value	< = 1 w	> 1w	*P* value
Overall	5489(61.06)	3501(38.94)		6328(70.39)	2662(29.61)	
Region			< 0.001			< 0.001
Shanghai	2738(72.28)	1050(27.72)		2971(78.43)	817(21.57)	
Fuzhou	1240(54.92)	1018(45.08)		1171(51.86)	1087(48.14)	
Chengdu	1511(51.32)	1433(48.68)		2186(74.25)	758(25.75)	
Education			< 0.001			< 0.001
Illiteracy and primary school	76(71.03)	31(28.97)		68(63.55)	39(36.45)	
Junior high school	800(77.07)	238(22.93)		745(71.77)	293(28.23)	
Senior high school	851(69.07)	381(30.93)		921(74.76)	311(25.24)	
University or junior college	3321(58.49)	2357(41.51)		3812(67.14)	1866(32.86)	
Master and above	441(47.17)	494(52.83)		782(83.64)	153(16.36)	
Occupation			< 0.001			< 0.001
Farmer	145(85.29)	25(14.71)		115(67.65)	55(32.35)	
Civil servant	93(63.27)	54(36.73)		117(79.59)	30(20.41)	
Teacher	147(44.95)	180(55.05)		242(74.01)	85(25.99)	
Medical staff	77(74.04)	27(25.96)		84(80.77)	20(19.23)	
White-collar	1310(62.95)	771(37.05)		1490(71.60)	591(28.40)	
Student	2210(54.92)	1814(45.08)		2702(67.15)	1322(32.85)	
Worker	497(68.74)	226(31.26)		493(68.19)	230(31.81)	
Freelance work	382(71.27)	154(28.73)		417(77.80)	119(22.20)	
Housework and unemployment	40(62.50)	24(37.50)		52(81.25)	12(18.75)	
Else	588(72.24)	226(27.76)		616(75.68)	198(24.32)	
Monthly Income in family (¥)			0.385			0.002
< 5000	1469(62.72)	873(37.28)		1571(67.08)	771(32.92)	
> = 5000 and < 10,000	1667(60.44)	1091(39.56)		1969(71.39)	789(28.61)	
> = 10,000 and < 20,000	1026(60.96)	657(39.04)		1201(71.36)	482(28.64)	
> = 20,000 and < 50,000	641(59.68)	433(40.32)		770(71.69)	304(28.31)	
> = 50,000	686(60.55)	447(39.45)		817(72.11)	316(27.89)	
Brand preference for vaccines			< 0.001			0.001
Specific preference	1522(64.19)	849(35.81)		1605(67.69)	766(32.31)	
No preference	3967(59.93)	2652(40.07)		4723(71.36)	1896(28.64)	
Vaccination hesitancy			< 0.001			0.005
Low	4167(67.90)	1970(32.10)		4385(71.45)	1752(28.55)	
Medium	1040(46.89)	1178(53.11)		1506(67.90)	712(32.10)	
High	282(44.41)	353(55.59)		437(68.82)	198(31.18)	
Domestic risk awareness			< 0.001			< 0.001
Low	3297(62.40)	1987(37.60)		3850(72.86)	1434(27.14)	
Medium	2030(58.55)	1437(41.45)		2307(66.54)	1160(33.46)	
High	162(67.78)	77(32.22)		171(71.55)	68(28.45)	
Disability			0.001			0.295
Yes	78(76.47)	24(23.53)		67(65.69)	35(34.31)	
No	5411(60.88)	3477(39.12)		6261(70.44)	2627(29.56)	
Contacted with GPs			< 0.001			0.056
Yes	400(68.73)	182(31.27)		430(73.88)	152(26.12)	
No	5089(60.53)	3319(39.47)		5898(70.15)	2510(29.85)	

#### Multivariate analysis

**Fig. 1:**
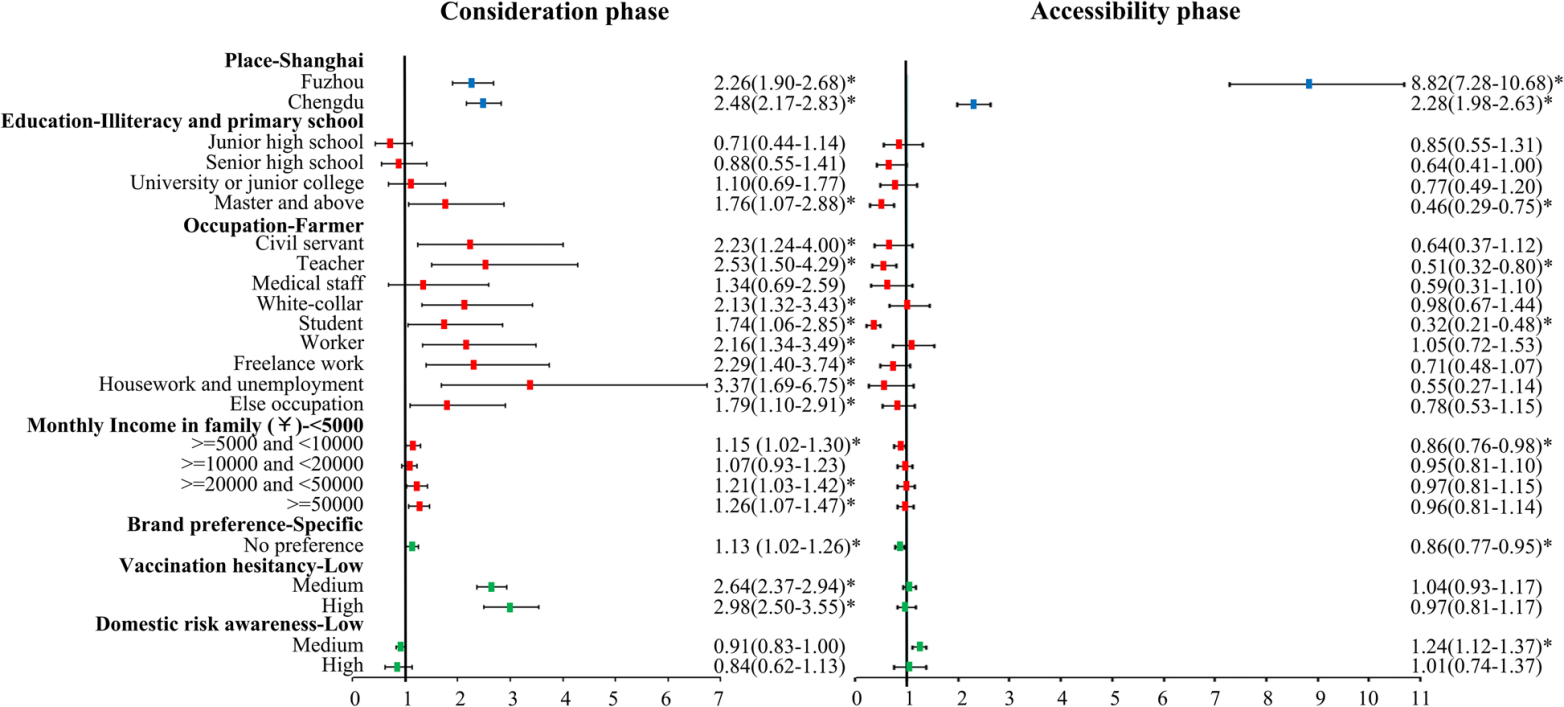
Multivariate analysis of factors associated with the two phases of the vaccination process. Binary logistic regression models were used to predict factors influencing the length of time categories to make an appointment and the length of time categories to receive a vaccination. The ‘*’ was representative for *p* < 0.05. Only the independent variables of the three dimensions (region, SES and personal attitudes towards COVID-19/vaccines) which are emphatically discussed in the study were represented in this figure. Covariates like disability and contacted with GPs were not presented

The logistic regression models (Fig. 1) included the following variables: region, SES (education, occupation, income), attitudes towards COVID-19 and vaccines (vaccine brand preference, vaccination hesitancy, risk awareness for the domestic epidemic), and other basic characteristics of participants (age, sex, disability, contacted with GPs). Above variables were tested and found to be significant in at least one phase. All two models were statistically significant (*p* < 0.05).

In the consideration phase (Fig. 1), the odds of an appointment decision taking longer than one month were 2.26 (95% CI: 1.90 to 2.68) times greater for participants in Fuzhou and 2.48 (95%CI: 2.17 to 2.83) times greater for participants in Chengdu than for participants in Shanghai. Moreover, such odds increased for participants with master and above degree compared with those who were illiterate or graduated from primary school (OR: 1.76, 95% CI: 1.07 to 2.88). Participants with higher monthly household incomes (reference category: < ¥5000) were also more likely to consider longer than one month. Occupational disparity was also significant. Compared with farmers, other professions, except for medical staff, were less likely to make an appointment within a month of hearing about COVID-19 vaccinations. For instance, the odds of the consideration phase being longer than one month were 3.37 (95%CI: 1.69 to 6.75) times greater for those engaged only in housework and for the unemployed than for farmers. Compared with participants with specific brand preference, the odds of the consideration phase being longer than one month were 1.13 (95%CI: 1.02 to 1.26) times greater for those without brand preference. The results also indicated that the odds of the consideration phase lasting more than a month increased with vaccination hesitancy (high hesitancy, OR: 2.98, 95%CI:2.50 to 3.55; medium hesitancy, OR:2.64, 95%CI:2.37–2.94; reference category: low hesitancy).

In the accessibility phase (Fig. 1), the odds of waiting longer than one week to receive a vaccination were 8.82 (95% CI: 7.28 to 10.68) times greater for participants in Fuzhou and 2.28 (95%CI: 1.98 to 2.63) times greater for those in Chengdu than for participants in Shanghai. Such odds decreased only for participants with master and above degree compared to those who were illiterate or graduated from primary school (OR: 0.46, 95% CI: 0.29 to 0.75). Compared with farmers, teachers (OR: 0.51, 95%CI: 0.32 to 0.80) and students (OR: 0.32, 95%CI: 0.21 to 0.48) were less likely to wait longer than one week. The significant influence of monthly household income (¥)was merely found in one category(> = 5000 and < 10,000, OR:0.86, 95%CI:0.76 to 0.98, reference category: < 5000). Meanwhile, participants without a brand preference (OR: 0.86, 95%CI: 0.77 to 0.95) were less likely to wait longer than a week after making an appointment to receive a vaccination. Moreover, this likelihood increased with higher risk awareness of a domestic epidemic (medium risk awareness, OR: 1.24, 95%CI:1.12 to 1.37; reference category: low risk awareness).

#### Regional differences in factors influencing the vaccination process

**Fig. 2:**
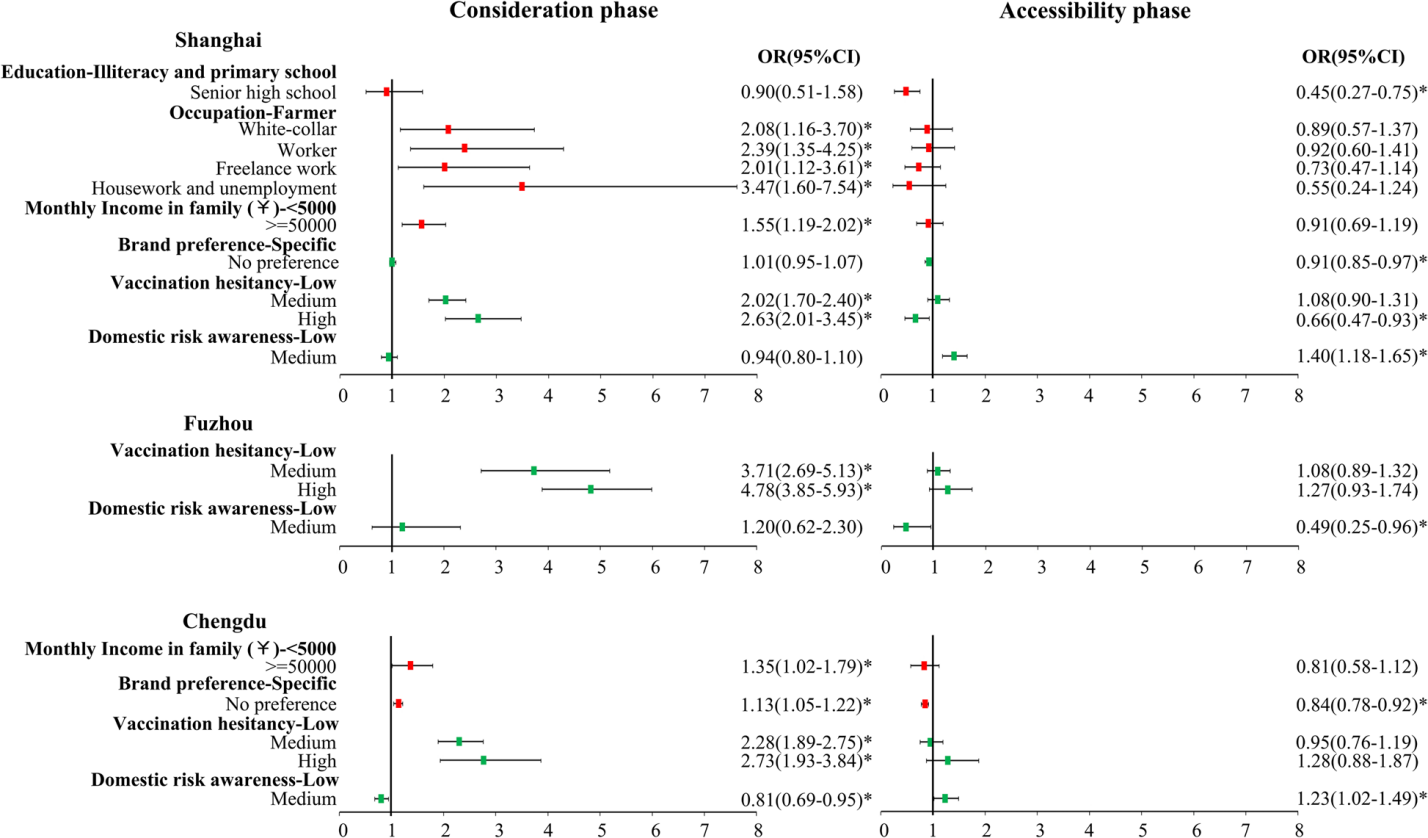
Subgroup analysis of three cities: Shanghai, Fuzhou, and Chengdu. All six models were statistically significant (*p* < 0.05). The ‘*’ was representative for *p* < 0.05. Variables included were the same in models for all three cities, while only variables that have at least one category that was significant in one or both phases are shown in the figure. Insignificant variables were not presented in the figure

The multiple subgroup factor analysis for the vaccination processes in Shanghai, Fuzhou, and Chengdu are shown in Fig. 2. The multi-variate models included following variables: SES (education, occupation, income), attitudes towards COVID-19 and vaccines (vaccine brand preference, vaccination hesitancy, risk awareness for the domestic epidemic), and other basic characteristics of participants (age, sex, disability, contacted with GPs). In Chengdu, no vaccine brand preference extended the consideration phase (OR:1.13, 95%CI:1.05 to 1.22, reference category: specific brand preference) but shortened the accessibility phase (OR:0.84, 95%CI:0.78 to 0.92). In Shanghai, the participants with no brand preference also tended to wait shorter in accessibility phase (OR:0.91, 95%CI:0.85 to 0.97). Participants graduating from senior high school were found to get vaccinated earlier after the appointment than those with lowest level of education (OR:0.45, 95%CI:0.27 to 0.75) in Shanghai. Higher household income in Shanghai and Chengdu and higher vaccination hesitancy in all three cities were significantly associated with longer consideration phase. Occupational disparities were found mainly in Shanghai. For example, house-based and unemployed participants were 3.47 (95% CI: 1.60 to 7.54) times more likely to have a longer consideration period than farmers.

## Discussion

In order to accelerate vaccination progress, this is the first study to account for the entire COVID-19 vaccination process, including both the deliberation period (consideration phase) and the waiting time to get vaccinated (accessibility phase). We found the consideration phase varied widely by region, SES, and attitudes toward vaccination, while the accessibility phase was less varied, indicating generally less inequality in this phase. These results indicated that the influential factors changed over different phases of the vaccination process, and it is significant to figure the differences out to promote vaccination process accurately.

Regional disparities about consideration and accessibility of vaccination among cities in mainland China were found in this study, which suggests the significance of primary care system in the vaccination campaign. Shanghai participants seemed to be more decisive in the period leading up to making a vaccination appointment and waited less time to get vaccinated compared with participants in Fuzhou and Chengdu, all of which is consistent with the actual vaccination process observed in China. Shanghai had administered more than 18 million COVID-19 vaccine doses as of May 10, 2021 [26], which was faster than all other cities in China; this could be attributed to the CHSCs, which acted as the vaccination site. There are 247 CHSCs in Shanghai, reported by the Shanghai Municipal Health Commission [27], and the number of health staff in Shanghai per thousand ranks second only to the capital, Beijing, among all cities in China. Thus, we believe that the relatively complete primary healthcare system in Shanghai enabled this city to be well-organized and implement a quick response. Regional disparities were observed in this study, a phenomenon that also prevails globally. The rate of COVID-19 vaccine doses administered per 100 people has ranged from 22 to 303 doses across countries [28]. With so many challenges to overcome in the rollout of COVID-19 vaccines worldwide [29], more action is needed, such as that taken by the program COVID-19 Vaccines Global Access (COVAX) [30], and we suggested a well-functioning primary care system is necessary in promoting equal access to vaccines.

Compared with regional differences, the effects of income and education on vaccination promotion were relatively limited. Participants with higher monthly household income and master or above degree tended to have a longer consideration phase compared with the lowest income (< ¥5000) or education level (illiteracy and primary school). People with higher education or income may think more carefully before deciding to get vaccinated. However, only one category of education and household income was associated with accessibility phase, disrupting the health disparity theory that higher income and education levels are associated with easier and faster access to better medical resources [31–33]. The relatively small impact of income and education on vaccine accessibility may be attributable to the COVID-19 vaccination plan in China, which has advocated that all residents make their vaccine appointments via an official online platform, and that all vaccinations are free at any CHSC. Such efforts to address barriers to vaccination and to achieve equitable access should be taken globally [34, 35]. Further, occupation had a significant effect especially in the consideration phase of Shanghai. In the whole sample, farmers were observed to be most decisive, while housewives and unemployed people were the most hesitant in the period before making an appointment. The quick decisions of farmers may be related to the vaccination promotion efforts in suburban areas, jointly promoted by village committees (a self-managed organization of rural residents) and GPs in village clinics (famous as barefoot doctor) [36], both of which are the most grassroots entities in suburban China. Housewives who care for children and adolescents may be more concerned about vaccine safety issues, while the unemployed participants are less likely to partake in collective mobilizations, especially the collectives offered by companies, the government, schools, and hospitals. In the second phase, teachers and students received their vaccinations more quickly after making an appointment compared with farmers as teachers and students were labeled as high-risk groups as they gather closely in classrooms and were provided with priority for vaccination. In case of Shanghai, China, population-targeted strategy to get vaccinated worked especially for densely populated commercial buildings and schools.

The impact of personal attitudes toward vaccines during the vaccination process was most mutable compared with SES and region. Vaccine brand preference promoted consideration but blocked accessibility compared with no brand preference in the whole sample. It is possible that those with a specific preference may have acquired more information about vaccinations in general; however, their preference for a brand may also have caused them to wait to get vaccinated until their preferred brand was available. In addition, hesitancy played a significant role in prolonging consideration process in all three cities. The negative impact of hesitancy in vaccination campaigns is consistent with most of the relevant literature [23, 37, 38]. In the case of China, a vaccination program promoted by multiple participants, including the government, GPs, village committees, and the media, proved effective in addressing the hesitation issue [39, 40]. These diverse strategies may have contributed to the relatively high acceptance rate of COVID-19 vaccinations in China compared with most countries included in previous studies [41, 42]. Our results showed that the medium level of domestic infectious risk awareness (reference: low domestic risk awareness) was associated with a longer accessibility phase, but played an insignificant role in the consideration phase. It is possible that participants are unwilling to gather in public places and get vaccinated in medical institutions when the domestic infectious risk is high. More importantly, new variants of COVID-19 and breakthrough infection cases may mitigate the public confidence in vaccines and lead to vaccine refusal [43].

There were some limitations in the current study that should be noted. Participants were recruited from three metropolitan areas in China. The vaccination process and associated factors were not explored for rural areas, which may differ widely from what we found in urban China. Thus, further research should be conducted in rural areas of China, especially remote areas that have not yet been studied. Moreover, in this observational study, our main focus was on the significant factors affecting the consideration and accessibility phases of the vaccination process, not on the underlying mechanisms of how those factors affect the vaccination process. Furthermore, specific interventions were not addressed in the present study. Future studies may investigate whether and how interventions associated with these factors can promote the vaccination process in a wider region, including both rural and urban areas. Finally, different cut-off times may change the results and conclusions. It would be better to adopt diverse cut-off times for analysis in future studies.

## Conclusions

This study found that the influential factors changed over the two phases of vaccination process. Regional disparity affected both the consideration and accessibility phases. Expect that, SES, and hesitancy were the major factors of the consideration phase, but had limited impact on the accessibility phase. These results suggest that a free and convenient vaccination plan for all, a well-functioning primary care system, a population-targeted strategy focusing on densely populated public places, and multiple-participating promotion are key elements to accelerate the vaccination process.

## Data Availability

The data generated and analyzed during the present study are only for academic use and are not publicly available because of privacy protection for participants. However, they are available from the corresponding author (jiaoling_huang@sina.com) for researchers who meet the criteria for access to confidential data.

## Abbreviations

COVID-19: Corona virus disease 2019
SARS-CoV-2: Severe acute respiratory syndrome coronavirus 2
SES: Socio-economic status
CHSCs: Community health service centers
GPs: General practitioners
COVAX: COVID-19 Vaccines Global Access
